# Awake Rat Brain Functional Magnetic Resonance Imaging Using Standard Radio Frequency Coils and a 3D Printed Restraint Kit

**DOI:** 10.3389/fnins.2018.00548

**Published:** 2018-08-20

**Authors:** Petteri Stenroos, Jaakko Paasonen, Raimo A. Salo, Kimmo Jokivarsi, Artem Shatillo, Heikki Tanila, Olli Gröhn

**Affiliations:** ^1^Kuopio Biomedical Imaging Unit, A.I.V. Institute for Molecular Sciences, University of Eastern Finland, Kuopio, Finland; ^2^Charles River Discovery Research Services Finland Oy, Kuopio, Finland

**Keywords:** awake imaging, fMRI, rat, BOLD, resting state, 3D printing, restraint kit

## Abstract

Functional magnetic resonance imaging (fMRI) is a powerful noninvasive tool for studying spontaneous resting state functional connectivity (RSFC) in laboratory animals. Brain function can be significantly affected by generally used anesthetics, however, rendering the need for awake imaging. Only a few different awake animal habituation protocols have been presented, and there is a critical need for practical and improved low-stress techniques. Here we demonstrate a novel restraint approach for awake rat RSFC studies. Our custom-made 3D printed restraint kit is compatible with a standard Bruker Biospin MRI rat bed, rat brain receiver coil, and volume transmitter coil. We also implemented a progressive habituation protocol aiming to minimize the stress experienced by the rats, and compared RSFC between awake, lightly sedated, and isoflurane-anesthetized rats. Our results demonstrated that the 3D printed restraint kit was suitable for RSFC studies of awake rats. During the short 4-day habituation period, the plasma corticosterone concentration, movement, and heart rate, which were measured as stress indicators, decreased significantly, indicating adaptation to the restraint protocol. Additionally, 10 days after the awake MRI session, rats exhibited no signs of depression or anxiety based on open-field and sucrose preference behavioral tests. The RSFC data revealed significant changes in the thalamo-cortical and cortico-cortical networks between the awake, lightly sedated, and anesthetized groups, emphasizing the need for awake imaging. The present work demonstrates the feasibility of our custom-made 3D printed restraint kit. Using this kit, we found that isoflurane markedly affected brain connectivity compared with that in awake rats, and that the effect was less pronounced, but still significant, when light isoflurane sedation was used instead.

## Introduction

Functional magnetic resonance imaging (fMRI) is a modern imaging modality enabling noninvasive measurements of neuronal activity. The detection is typically based on the blood oxygenation-level dependent (BOLD) contrast (Ogawa et al., 1990), which exploits the neurovascular coupling cascade to provide an indirect measure of brain activity. Traditionally, fMRI is used to detect local activation changes induced by a precise stimulus, but advanced fMRI techniques, such as resting-state fMRI (Biswal et al., 1995), have enabled studies of resting state functional connectivity in large-scale brain networks.

Despite the fMRI method being fully compatible with clinical settings, experimental rodent studies are still irreplaceable, as they not only provide a means to scrutinize the neurovascular coupling mechanisms, but also allow for controlled genetic, electrical, and pharmacologic manipulations, and more invasive monitoring that enables mechanistic studies of normal and abnormal brain functions. In the majority of preclinical fMRI studies, however, anesthetics complicate interpretations of the results. Anesthesia is required to prevent the animals from moving as well as to decrease discomfort and stress in the animals, but it also has significant undesirable effects on fMRI responses to stimuli or drugs (Paasonen et al., 2016, 2017) and resting state activity (Hamilton et al., 2017; Paasonen et al., 2018) by interfering with normal brain function and neurovascular coupling (Franks and Lieb, 1994; Martin et al., 2006; Zhao et al., 2007; Ferron et al., 2009; Gao et al., 2016).

To avoid the disrupting effects of anesthetics, several groups have implemented awake animal fMRI imaging protocols (King et al., 2005; Zhang et al., 2010; Becerra et al., 2011; Febo, 2011; Tsurugizawa et al., 2012; Brydges et al., 2013; Chang et al., 2016; Kenkel et al., 2016). Awake animals are likely to move and experience stress during fMRI, which raises the demand for carefully optimized restraint and habituation protocols. Several studies have demonstrated, based on physiologic markers of stress, that rats are able to adapt to the level of restraint required for fMRI (King et al., 2005; Tsurugizawa et al., 2010; Reed et al., 2013; Chang et al., 2016).

Despite the advantages provided by awake animal imaging, the method is still relatively rarely exploited, partly due to several methodologic issues. First, there are only a few readily available solutions for restraint implementation, leading to a demand for custom-made restraint kits and beds. Second, the custom-made restraint parts are typically not compatible with standard radiofrequency coils. If large volume coils are used for signal reception, decreases in the signal-to-noise ratio hinder the detection of fMRI signal changes. Third, the habituation protocols can last up to 8 days (King et al., 2005) making the approach very time-consuming. Therefore, there is a clear demand for fast, easily implemented, low-stress, and low-cost approaches that could be performed using standard hardware available in the preclinical MRI laboratory.

To address some of the essential methodologic limitations in awake rat imaging, the first aim of this study was to construct an open-access 3D printable restraint kit that is fully compatible with a standard Bruker rat bed and surface coil. The second aim of this study was to investigate the possibility of performing a habituation protocol under very light sedation that would minimally affect brain function, but at the same time allow for a short habituation period. The developed restraint kit and habituation protocol were implemented into fMRI studies to obtain RSFC data from awake and lightly sedated (0.5% isoflurane) rats, and the RSFC data were subsequently compared with corresponding data obtained from anesthetized (1.3% isoflurane) rats.

## Materials and methods

### Restraint parts

**Figure 1:**
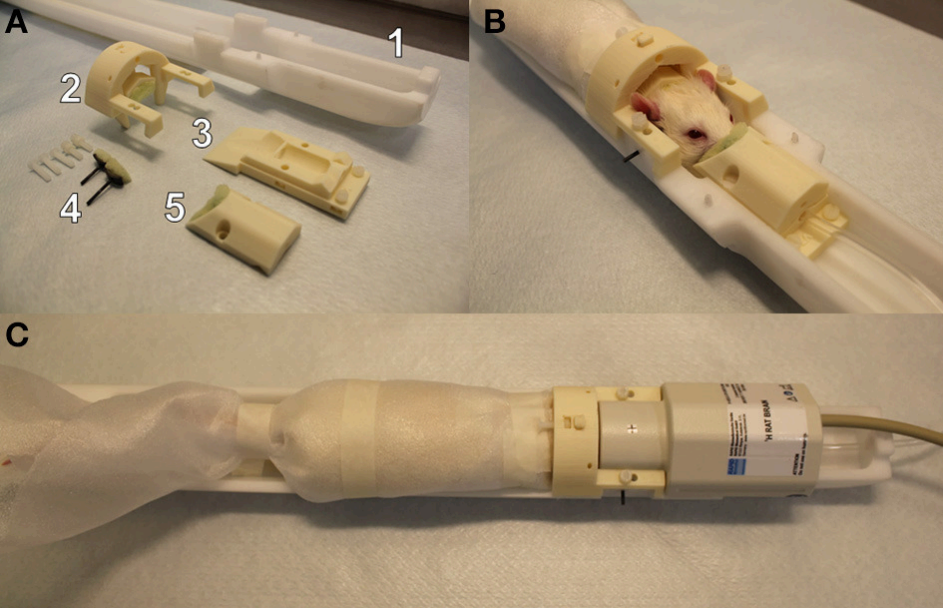
A custom-made 3D printed rat restraint kit **(A)**, a rat restrained in the bed with the developed restraint kit **(B)**, and a restrained rat in the bed with a Bruker quadrature receiver coil attached **(C)**. The rat restraint kit **(A)** consists of a standard Bruker rat bed **(A1)**, a padded shoulder/neck support **(A2)**, a sliding sledge including a bite bar **(A3)**, padded cheek supports **(A4)**, and a padded nose cone **(A5)**. A replicate of Bruker bed was used in mock scanner.

The custom-made 3D printed (with Zmorph 2.0 SX) restraint kit (Figure 1), compatible with an existing Bruker Pharmascan rat bed and quadrature surface receiver coil (Bruker Biospin, Ettlingen, Germany), was designed for rat brain imaging. The restraint kit, printed from acrylonitrile styrene acrylate (other materials e.g., acrylonitrile butadiene styrene can be also used), comprises a foam-padded arc-shaped shoulder and neck support, padded cheek supports, and a sliding sledge with a padded nose cone, which functions as a gas mask. The sliding sledge also includes a bite bar. Thus, the contact points for the restraint are neck area, shoulders, muzzle and cheeks of the rat. The RF-coil is attached on top of the head making little physical contact.

The 3D printed restraint kit was designed with CAD software (Autodesk 123D Design), and the kit requires no additional modifications (e.g., screw threads) to the animal bed. The 3D printing files (file format: STL), excluding the MRI bed, are available in the electronic Supplementary Material. For the cheek supports, we provide a 3D printable model for the whole support and separately for a curved plate (presses against the cheek) but we recommend to use a tougher carbon fiber bar that can be fixed to the plastic plate. In 3D printing, we used layer thickness of 200 μm for all parts except for the cheek support bars 50–100 μm layer thickness is recommended. Also, we advise to use support material in 3D printing. In addition to 3D printed parts, plastic screws (diameter 3.8 mm, length 20 mm) and compatible plastic nuts (outer diameter 7 mm) were used for attaching the restraint parts, and foam pad was used to soften points of restraint.

### Animals

All animal procedures were approved by the Animal Experiment Board in Finland, and conducted in accordance with the guidelines set by the European Commission Directive 2010/63/EU.

A total of 23 adult male rats were used (RccHan Wistar, 320 ± 25 g, purchased from Laboratory Animal Centre, University of Eastern Finland). Rats were divided into three groups: awake (*n* = 8), lightly sedated (isoflurane 0.5%, *n* = 7), or anesthetized (isoflurane 1.3%, *n* = 8). The rats in the 1.3% isoflurane group were group-housed in cages, while rats in the awake and lightly sedated groups were individually housed because the rats were also individually habituated. All animals were maintained on a 12/12 h light-dark cycle at room temperature of 22 ± 2°C with humidity of 50–60%. Food and tap water were available *ad libitum*.

### Habituation and imaging schedule for awake and lightly sedated rats

**Figure 2:**
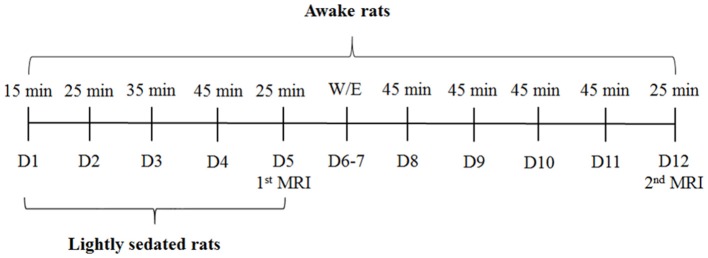
Rat habituation and imaging schedule. D, days, W/E, weekend.

Habituation sessions were gradually increased from 15 to 45 min (Figure 2). Rats in the lightly sedated group were habituated for 4 consecutive days prior to fMRI. Rats in the awake group were habituated and imaged in two 5-day periods. During the first week, we used a protocol identical to that for the lightly sedated group. In the following week, training was continued for another 4 days with fixed 45-min sessions, followed by a second fMRI session. Rats were habituated daily and imaged (two rats sequentially at one session) at the same time of day (9.00–12.00 a.m.) to control for circadian rhythm variations.

### Habituation protocol for awake and lightly sedated rats

Rats were habituated for imaging in a mock scanner mimicking the real MRI environment. Located in a separate habituation room, the mock scanner comprised a plastic tube (ID = 72 mm) mimicking a magnet bore, a rat bed, a standard platform for the rat bed, a web camera for recording movement, and a speaker (JBL LSR308, Harman International Industries, Stamford, CT, USA). We measured the sound level using a microphone (MT830R, Audio-Technica, Leeds, England) positioned inside the real MRI bore and played the sounds of the MRI at the same sound pressure as that inside an actual animal scanner.

At the beginning of each habituation session, the rats were first anesthetized with 5% isoflurane (Baxter, Lessines, Belgium; in a carrier gas mixture of N_2_/O_2_ [70/30]), weighed, and transferred to a hood where anesthesia was maintained with 1.5–2.0% isoflurane. The restraint protocol described below is also available in video format (Supplementary Video 1). A piezoelectric pneumatic sensor (M2M, Cleveland, OH, USA) was taped onto the belly and adhesive electrocardiography (ECG) electrodes (3M, St. Paul, MN, USA) were secured to the limbs. The forepaws were attached with masking tape along the sides of the rat without allowing the adhesive surface of the tape to come into contact with the rat fur. The hind paws were tied together with the tail with masking tape.

A sheet of foam plastic was wrapped around the rat from the shoulders to the hind paws to restrain body movement. Foam plastic assured a tight, but flexible and warm environment that allowed the rat to breathe normally. Foam plastic was secured with tape around the shoulders and hind paws. Silicone plugs were used to protect hearing from the MRI scanner sounds, thus increasing comfort.

Next, the rat was transferred to the animal bed in the mock scanner. Isoflurane was maintained at 1.5–2.0%. The head was placed on the sliding sledge and the upper teeth were secured behind the bite bar. The nose cone was tightened to the sledge to prevent muzzle movement in the vertical direction. The padded cheek supports, positioned on the both sides of the head, were gently tightened below the ears to prevent lateral movement of the head. The head was further secured by adjusting the shoulder and neck support; this also reduces the effects of lower body movements on the head. The nose cone, cheek supports, and shoulder/neck support also suppressed rotational movement of the head. To minimize body movement, tape was placed around the rear part of the body and attached to the bed.

ECG electrodes and a breathing sensor were connected to an amplifier (Biovet CT1 system, m2m imaging, Corp., USA). A heating pad was placed on top of the rat and connected to a Biovet CT1 temperature regulation unit to keep the body warm. To minimize discomfort, a rectal probe was not used but thermometer was placed under the animal to monitor temperature.

Finally, a Bruker rat surface quadrature receiver coil was placed on top of the head, and the animal bed was pushed inside the mock MRI tube. The receiver coil used in the habituation sessions was similar to the one used during the actual fMRI session.

The pre-habituation preparations lasted approximately 8 min per rat, excluding the induction of anesthesia. After the preparations, isoflurane was decreased to either 0.5% in the lightly sedated group or turned off in the awake group.

MRI sounds, starting with the sounds of pilot imaging and shimming, and then continuing with continuous EPI scanning, were played for a given time (15–45 min, see Figure 2), while simultaneously monitoring the physiologic parameters, i.e., the respiratory rate and heart rate. Movement was monitored and recorded through a web camera. If the rat was moving excessively, defined as continuous movement >30 s, or more than a third of the time used for the session, the habituation protocol was ceased and continued the next day.

After reaching the desired habituation session length (see Figure 2), isoflurane was raised to 2%. When physiologic parameters indicated a sufficient depth of anesthesia, the rat was released from the kit and transferred back to the hood where the plastic foam and tape were removed. Blood samples (total of 150 μl) were obtained from the lateral tail vein. Next, the rat was returned to the cage and chocolate cereal balls were given as a reward. Blood samples were centrifuged at 3,500 RPM for 10 min, after which plasma was collected and samples were stored at −70°C for corticosterone analysis.

### fMRI protocol

MRI was performed with a 7T/16cm horizontal Bruker Pharmascan system and ParaVision 5.1 software. A standard Bruker quadrature resonator volume coil (ID = 72 mm) and a rat brain quadrature surface coil were used. A 3D field-map based shimming method was used to optimize the field homogeneity.

Functional imaging was performed with single-shot spin-echo echo planar imaging sequence with the following parameters: repetition time 2,000 ms, echo time 45 ms, matrix size 64 × 64, field-of-view 2.5 × 2.5 cm, 9–11 slices of 1.5 mm thickness, and a bandwidth of 250 kHz. The same imaging parameters were used with all animals, except that 300 fMRI volumes (10 min) were acquired from anesthetized rats and 600–750 fMRI (20–25 min) volumes were acquired from awake and lightly sedated rats. Despite habituation, awake animals tend to move slightly. Therefore, a longer period was obtained from awake and lightly sedated rats to ensure a continuous motion-free 10-min period for analysis.

Anatomic images were acquired after the fMRI scans with fast spin-echo sequence with the following parameters: repetition time 4,680 ms, echo spacing 16.1 ms, 8 echoes, effective echo time 48.4 ms, matrix size 512 × 512, field-of-view 5.0 × 5.0 cm, 30 slices of 0.75 mm thickness, and bandwidth of 46.875 kHz. The restraint preparations for the awake and lightly sedated rats were similar to those for the habituation protocol, except that heart rate was measured using a pulse oximetry sensor.

Physiologic parameters (heart rate, respiration, and temperature) were monitored continuously during the imaging, and movement was estimated from the real-time reconstructed EPI images. Rats were kept warm using a water circulation heated animal bed. After measurements, blood samples for corticosterone level analysis were collected as in the habituation sessions.

The protocol for imaging anesthetized rats (isoflurane 1.3%) was describer earlier (Paasonen et al., 2018). Briefly, small cannulas (BD Intramedic™ PE-10, Franklin Lakes, NJ, USA) were inserted into the femoral artery and vein, and tracheostomy was performed under 2% isoflurane anesthesia. As isoflurane anesthesia suppresses respiratory function and easily leads to hypercapnia, mechanical ventilation (Inspira, Harvard Apparatus) was used to maintain normal blood gas values (pCO_2_ 45.1 ± 2.0; i-STAT Model 300, Abbott Point of Care Inc., Princeton, NJ, USA) measured from the arterial blood sample (150 μl). Muscle relaxant (~1 mg/kg/h i.v., pancuronium bromide, Pavulon (R), Actavis) was given while connecting the animal to the ventilator. Rats were killed immediately after the measurements.

### fMRI analysis

First, a 10-min motion-free fMRI data period was selected from each subject. Next, the fMRI data were converted to NIfTI (http://aedes.uef.fi), slice-timing corrected, motion-corrected, spatially smoothed, and co-registered to a reference brain (SPM8).

Regions of interest (ROIs) were drawn according to an anatomic atlas (Paxinos and Watson, 1998). The 12 ROIs for whole brain analysis were the medial prefrontal cortex (mPFC), motor cortex (MC), somatosensory cortex (SC), visual cortex (VC), auditory cortex (AC), retrosplenial cortex (RC), nucleus accumbens (NAc), striatum (Str), hippocampus (HC), medial thalamus (ThM), ventrolateral thalamus (ThVL), and hypothalamus (HTH) (Supplementary Figure 1A).

Additional ROIs were drawn for the default mode network (DMN) analysis, based on the article by Lu et al. (2012): medial frontal cortex (including prelimbic cortex and anterior cingulate cortex), temporal association cortex, orbitofrontal cortex, auditory cortex, retrosplenial cortex, posterior parietal/visual cortex and hippocampus (Supplementary Figure 1B).

RSFC was estimated with ROI-based Pearson correlation (r) analysis. Prior to correlation calculations, data were band-pass filtered at 0.01–0.15 Hz. Subsequently, the *r*-values were transformed to Z-scores prior to calculating group averages and statistical comparisons. After performing the calculations, the mean Z-scores were returned to *r*-values. Correlation coefficients between the 12 ROIs were used to form functional connectivity (FC) matrices (Figure 4) and the same ROIs were used as seed regions in correlation maps. Voxel-wise correlation maps for the awake group were calculated by using somatosensory cortex, striatum, and medial thalamus as seed regions (Figure 5A). Subsequently, difference maps between the awake and 0.5 and 1.3% isoflurane groups were calculated (Figure 5B). Mean correlation values (Supplementary Figure 2) from each of the 12 ROIs were additionally calculated to obtain mean connectivity of the brain regions. Correlation coefficients within the rat DMN were also calculated (Figure 6). Moreover, we calculated partial correlation coefficients by using mass center displacement values (x, y, z and roll, pitch, yaw) as a regression for motion.

Statistical group-level differences (*p* < 0.05) in DMN and mean functional connectivity were calculated using a one-way ANOVA and Tukey's multiple comparison *post-hoc* test. FDR-corrected *t*-tests (*p* < 0.05) were applied to FC matrices. FDR-corrected, one-sample *t*-test (*p* < 0.001) was performed to obtain functional connectivity map from the awake rats. Two-sample *t*-test (*p* < 0.05) was performed to compare 0.5 and 1.3% isoflurane groups to the awake rats. Statistical testing was performed with GraphPad Prism (Version 5.03, GraphPad Software Inc., La Jolla, CA, USA) or Matlab (R2011a), Natick, MA, USA.

### Analysis of movement during fMRI

In addition to visually estimated movement incidents, motion correction parameters, obtained from SPM8, were analyzed to evaluate the movement of the head during the fMRI session. Translational (x-y-z-directions) and rotational (roll, pitch, and yaw) movements were calculated.

First, co-registered translational and rotational mass center displacement values, given by SPM8, were detrended and normalized. Movement was evaluated from these values during the awake fMRI session by subtracting every subsequent time-point from the preceding value. After removing visible movement incidents (sharp peaks in mass center displacement) from a selected 10-min fMRI period, we preprocessed the data again to obtain 10-min motion-free fMRI periods. In these periods the motion was substantially lower in relation to voxel size (Supplementary Figure 3A). Of 16 awake datasets, 2 (2/16) were excluded from the analysis due to excessive movement.

Each subtracted translational and rotational movement was then converted to absolute values. These absolute values were summated over 10-min MRI sessions to obtain summated translational and rotational movement (Supplementary Figure 3B). Additionally, absolute maximum translational displacement in the y-direction, in which most of the movement happened, was calculated to estimate overall displacement of the animal head in the restraint kit (Supplementary Figure 4). This displacement value represents maximum displacement of the animal head from the starting position and does not inform of the separate movement incidents.

### Estimation of stress

Physiologic values (plasma corticosterone level, respiratory rate, heart rate, movement, and weight) were monitored during the habituation and fMRI sessions to estimate stress. Plasma corticosterone concentrations were quantified by a corticosterone immunoassay (Corticosterone rat/mouse ELISA Cat. No.: RTC002R, Demeditec Diagnostics, Kiel, Germany). Corticosterone concentrations were measured with a microtiter plate reader (Labsystems Multiskan MS, Vantaa, Finland) at an absorbance of 405 nm. Average respiratory and heart rates were calculated from values during habituation and fMRI sessions. Only values from 6 min onwards were included to minimize the possible influence of anesthesia on arousal. If the interval between two visually estimated movement indicents was ≥4 s (two fMRI volumes), they were considered separate incidents.

Additionally, we estimated the potential habituation-induced depression and anxiety by conducting open-field and sucrose preference behavioral tests with a small subgroup of awake habituated rats (*n* = 5). Control rats (*n* = 5) were anesthetized daily with isoflurane for the same amount of time that the habituated rats were anesthetized.

In the open-field test we used a circular plastic arena (ID = 100 cm) placed in a quiet room. Color of the arena was black to distinguish white Wistar rats from the environment. We used a lux meter (TR-710, Trifitek Finland Oy, Alajärvi, Finland) to confirm homogenous lighting conditions (19.3 ± 1.1 lx, 9 measurement points) across the test arena. Test was performed 10 days after the last procedure. In the test, rat was placed on the arena edge having its nose toward the wall. Movement and other activities were video-recorded for 10 min. Data were analyzed with Ethovision XT 7.1 (Noldus, Wageningen, The Netherlands), where the arena was divided into center (circle with 40 cm diameter) and edge (up to 10 cm from borders) zones. Distance traveled, activities (rearing and grooming), and time spent in different zones were subsequently calculated.

In the sucrose preference test, rats were first habituated to 1% sucrose water, which was made available *ad libitum* for 2 days next to regular tap water. During the next 2 days, the bottles with sweetened and regular water were weighed to calculate consumption during each day. The positioning of the bottles was switched each day.

The plasma corticosterone level, respiratory rate, heart rate, movement, and weight measurements were analyzed with ANOVA for repeated measures and Dunnett's *post-hoc* test in GraphPad Prism. Statistical comparison of corticosterone levels between days was done by comparing each day to baseline level. Comparison of respiratory rate, heart rate, movement and weight between days were done by comparing each day to habituation day 1. Statistical testing for the open field and sucrose preference tests was performed with a two-sample, two-tailed *t*-test assuming equal variances.

## Results

### Stress level indicators

**Figure 3:**
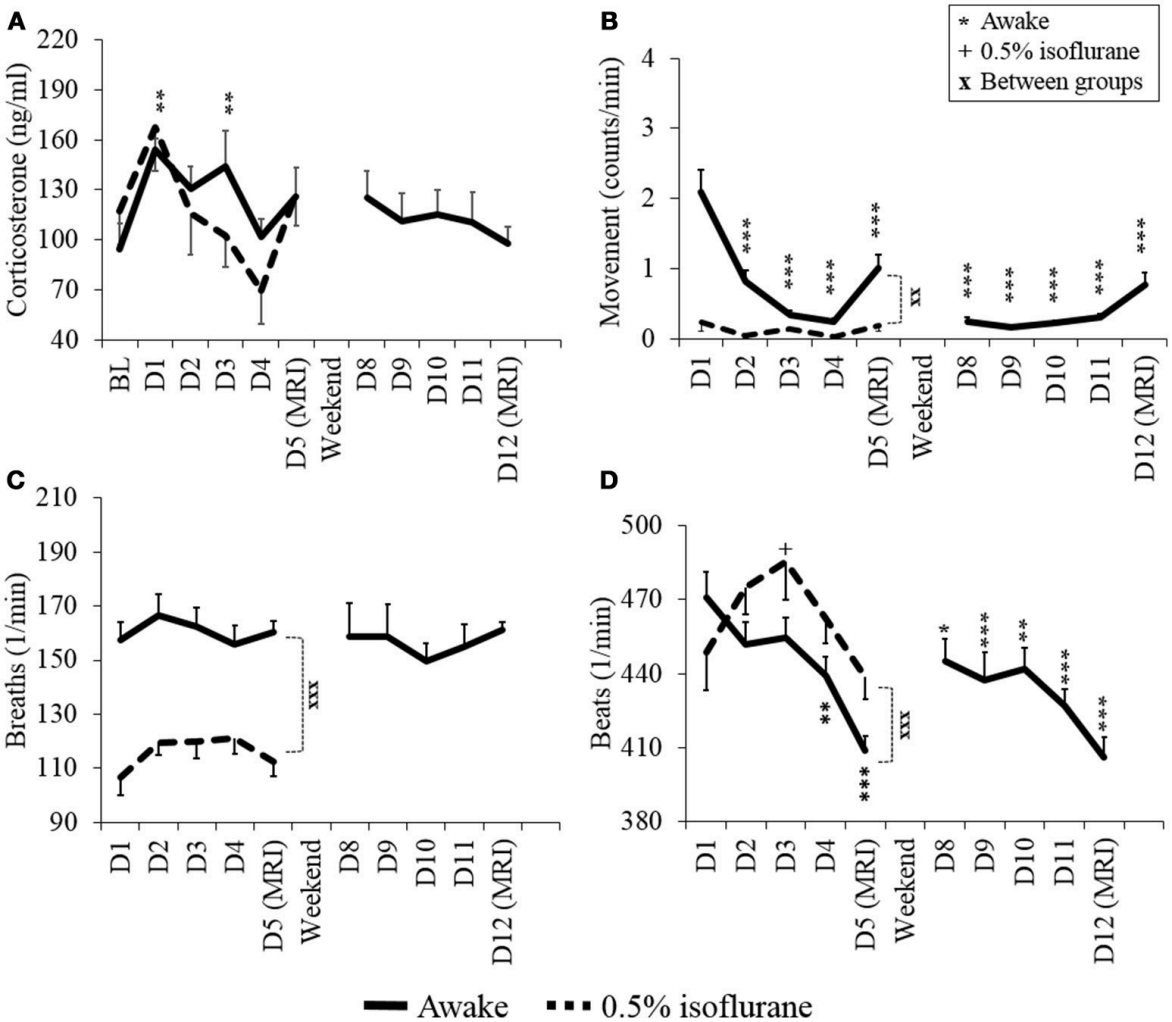
Stress level indicators of plasma corticosterone **(A)**, movement **(B)**, respiratory rate **(C)**, and heart rate **(D)** of awake and lightly sedated rats during habituation and fMRI. Mean values ± SEM of corticosterone (ng/ml), movement (counts/min), respiration (breaths/min) and heart rate (beats/min) are presented (*n* = 7–8 in each group). Statistical testing between days was done with ANOVA for repeated measures and Dunnett's *post-hoc* test, and between groups with a two-sample *t*-test. Significant differences of each time point are co mpared to either BL **(A)** or D1 **(B–D)** values. One symbol equals *p* < 0.05, two symbols equal *p* < 0.01, three symbols equal *p* < 0.001.

The measured values for stress level indicators (plasma corticosterone level, heart rate, respiratory rate, and movement) obtained from awake and lightly anesthetized rats during the habituation protocol are summarized in Figure 3.

Plasma corticosterone concentrations on the MRI day 1 did not differ statistically from the baseline concentrations in the awake or lightly sedated groups (*p* = 0.25 and *p* = 0.36, respectively, paired *t*-test). In the awake group, corticosterone concentrations significantly increased from the baseline level on habituation day 1 (*p* = 0.014, paired, *t*-test). However, concentrations returned to baseline level on the habituation day 4 (baseline: 95 ± 43 ng/ml, day 4: 102 ± 30 ng/ml, *p* = 0.75, paired, *t*-test). Also corticosterone concentration did not appear to differ statistically on MRI day 2 from MRI day 1 (*p* = 0.064, paired *t*-test). Noticeably, in the lightly sedated group, there was no clear difference between the baseline (117 ± 20 ng/ml) or the 1st habituation day (168 ± 27 ng/ml) (*p* = 0.066, paired *t*-test).

Movement of awake rats decreased significantly already during habituation day 2 (*p* = 0.002, paired *t*-test) and, from thereon, differed significantly in each habituation and MRI days from habituation day 1. Importantly, there was no further decrease in movement on MRI day 2 (0.77 ± 0.18 counts/min) compared with MRI day 1 (1.0 ± 0.20 counts/min) (*p* = 0.20, paired *t*-test). In the lightly sedated group, movement did not differ significantly between days but rats moved significantly less during MRI day 1 compared to awake rats (*p* < 0.001, two-sample *t*-test).

The heart rate of awake rats was significantly lower during habituation day 4 and MRI day 1 compared with habituation day 1 (*p* = 0.018 and *p* < 0.001, respectively, paired *t*-test). Heart rates during MRI day 2 did not differ significantly from MRI day 1 (*p* = 0.67, paired *t*-test). In the lightly sedated group there was no significant difference in the heart rates between habituation day 1 and MRI day 1 (*p* = 0.49, paired *t*-test).

The breathing rate was significantly lower during MRI day 1 in lightly sedated group compared with the awake group (*p* < 0.001, two-sample *t*-test) but the levels did not change significantly in either group between the habituation days. Additionally, average body weight of animals remained stable during the habituation (varied between 316 to 325 ± 4.2 g in awake rats and 319 to 320 ± 5.1 g in lightly sedated rats) (data not shown).

Results from the open field and sucrose preference tests are illustrated in the Supplementary Figures 5, 6. There were no differences in locomotor activity, time spent in different zones, rearing activity, or sucrose preference between the groups (*p* > 0.05, two-sample *t*-test). However, habituated rats spent more time grooming than control rats (20.2 ± 3.6 s vs. 4.9 ± 2.8 s, *p* = 0.01, two-sample *t*-test).

### Movement during fMRI

Generally, the awake rats moved more compared with the other groups. For example, there was a significant difference in the summated movement in X- (1st awake time-point 988 μm, 1.3% group 339 μm, *p* < 0.001, two-sample *t*-test) and Y- (1st awake time-point 3,330 μm, 1.3% group 423 μm, *p* < 0.001, two-sample *t*-test) directions, and in rotational directions of roll (1st awake time-point 18.3°, 1.3% group 11.0°, *p* < 0.01, two-sample *t*-test) and yaw (1st awake time-point 9.11°, 1.3% group 3.40°, *p* < 0.001, two-sample *t*-test) between the 1st awake time-point and 1.3% isoflurane groups. Maximum displacement from the mass center was also significantly higher in the 1st (253 μm) and 2nd (271 μm) awake time-points compared with the 1.3% isoflurane group (51 μm; *p* < 0.001 and *p* < 0.01, respectively, two-sample *t*-test). Notably, lightly sedated rats did not move significantly more than anesthetized rats based on summated movement or displacement values. Movement incidents, summated movement and displacement values during 10-min fMRI sessions are illustrated in Supplementary Figures 3 and 4.

### Resting state functional connectivity

#### Functional connectivity matrices

**Figure 4:**
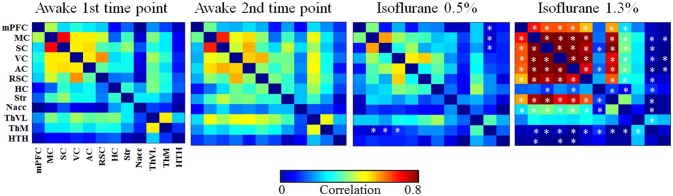
The group-level functional connectivity (FC) obtained from the 1st (*n* = 8) and 2nd (*n* = 6) awake time points, lightly sedated rats (*n* = 7), and anesthetized rats (*n* = 8). The connectivity data were analyzed from 300 volumes from 12 regions of interests. Statistical testing was done by using FDR-corrected *t*-test, *p < 0.05, **p < 0.01, ***p < 0.001. Significant differences against 1st awake time point are presented. AC, auditory cortex; HC, hippocampus; HTH, hypothalamus; mPFC, medial prefrontal cortex; ThM, medial thalamus; MC, motor cortex; Nacc, nucleus accumbens; RSC, retrosplenial cortex; SC, somatosensory cortex; Str, striatum; ThVL, ventrolateral thalamus; VC, visual cortex.

Functional connectivity matrices are shown in Figure 4. FC matrices obtained from the awake animals after the first and second habituation week were almost identical with no significant difference between them (*p* > 0.99, *t*-test, FDR adjusted). In the lightly sedated group, connectivity was significantly weaker in the three pathways compared with that in the first awake group: between medial thalamus and motor, somatosensory, and visual cortex.

In the anesthetized group, correlation values were significantly higher in several (21/66) cortico-cortical and cortico-striatal connections compared with the first awake group (*p* < 0.05, *t*-test, FDR adjusted). In addition, statistically stronger connectivity in anesthetized rats was observed in mean connectivity values in the medial frontal cortex (0.41), motor (0.61), somatosensory (0.60), visual (0.59), auditory (0.57) cortices, and in the striatum (0.49) compared with the first awake group (medial frontal cortex [0.20], motor [0.37], somatosensory [0.37], visual [0.37], auditory [0.33] cortices, and striatum [0.26]) (*p* < 0.05, Supplementary Figure 2).

In contrast, the correlation was significantly lower in several subcortical-cortical (22/66) connections in anesthetized rats compared with the awake rats (*p* < 0.05, *t*-test, FDR adjusted). Further, the mean correlation was significantly lower in the medial thalamus (*p* < 0.01) in anesthetized rats than in awake rats.

Seed-based partial correlation, with motion as a regression, did not differ statistically from correlation values calculated without motion regression (*p* > 0.05 *t*-test, FDR adjusted). Therefore, the possible remaining motion artifacts were not affecting the results obtained with functional connectivity analyses.

#### Voxel-wise correlation maps

**Figure 5:**
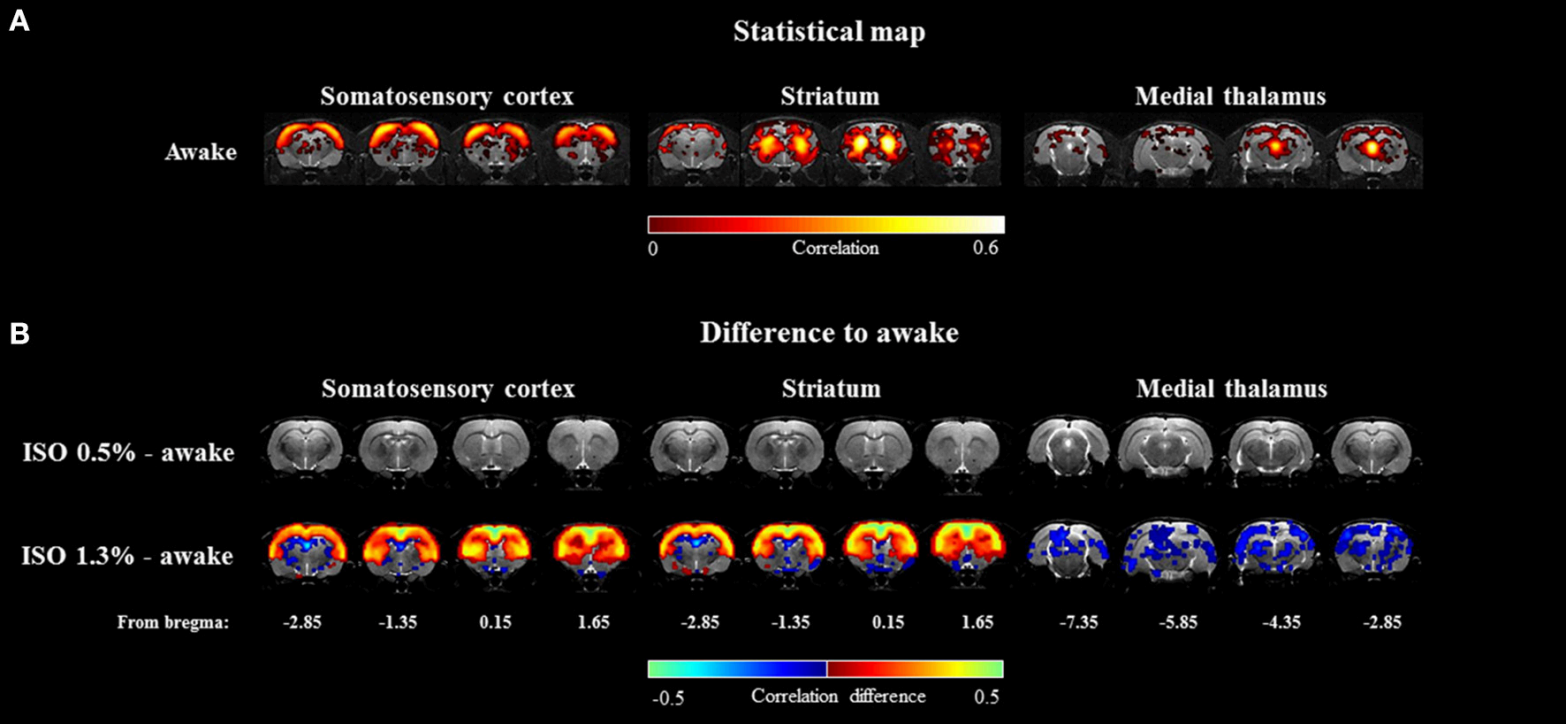
The group-level seed-based statistical correlation maps obtained from the awake group **(A)** and group-level differences compared to the awake group **(B)** obtained from three representative brain regions. Brain regions of somatosensory cortex, striatum, and medial thalamus were selected. Statistically significant voxels were obtained with one-sample *t*-test **(A)** (*p* < 0.001) and two-sample *t*-test **(B)** (*p* < 0.05).

The seed-based mean correlation map and group difference maps obtained from three representative ROIs are shown in Figure 5. In the 1.3% isoflurane group, the connectivity of the somatosensory cortex and the striatum was more widespread and stronger compared to the awake group, while thalamo-cortical connectivity was clearly suppressed. In the lightly sedated group, we did not find statistically significant voxel-wise correlation differences compared to the awake group after multiple comparison correction.

#### Default mode network

**Figure 6:**
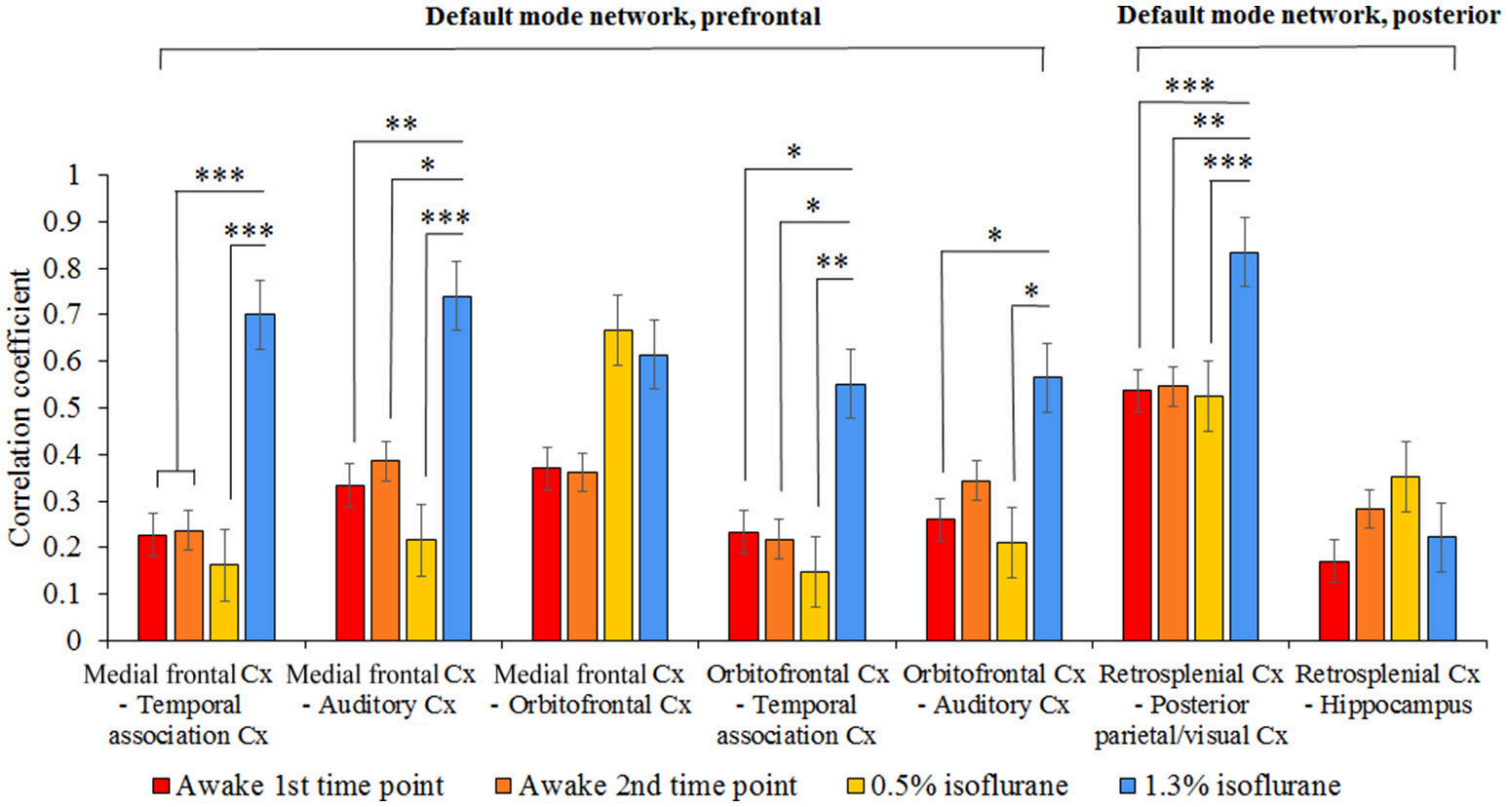
The group level mean correlation coefficients (± SEM) between the seven pathways in the rat DMN. The 300-volume data (10 min) were obtained under two awake time points, lightly sedated and anesthetized conditions. Statistical testing was done by one-way ANOVA and Tukey's multiple comparison post-hoc test, *p < 0.05, **p < 0.01, ***p < 0.001. Cx, cortex.

The functional connectivity of seven pathways in the rat DMN is illustrated in Figure 6. In the 1.3% isoflurane group, connectivity was stronger in almost all DMN connections (*p* < 0.05), excluding medial frontal–orbitofrontal and retrosplenial cortex–hippocampus connections, when compared with the first awake time-point. In the lightly sedated group, the DMN connectivity did not differ statistically from those in the awake groups.

## Discussion

It has become increasingly recognized that anesthesia is a major confounding factor in animal fMRI studies, especially when assessing functional connectivity in the brain (Liang et al., 2012, 2015; Liu and Duyn, 2013; Paasonen et al., 2018). Awake animal imaging offers the possibility of avoiding the drawbacks of anesthesia, and robust, practical, and low-stress awake imaging methods are in high demand. Indeed, a number of different approaches are available for awake fMRI (Table 1).

**Table 1:** Different rat restraint and habituation methods from literature.

**Publications**	**Species**	**Type of MRI receiver coil**	**Restraint of the head**	**Restraint of the body**	**Habituation protocol**	**Padded holder parts**	**Ear bars**	**Ear plugs**	**Corticosterone levels**	**MRI applications**
**Current study**	**Wistar**	**Quadrature surface coil**	**3D printed restraint kit compatible to standard rat beds and coils**	**Elastic plastic foam**	**15–45 min with increments of 10 min/day, 4–9 days**	**Yes**	**No**	**Yes**	**Decrease in corticosterone, 126** ± **18 ng/ml after fMRI (D5)**	**Resting state fMRI**
King et al., 2005	Sprague-Dawley	Single loop, surface coil	Cylindrical head holder	Body tube	90 min/day, 4–8 days	No, but topical analgesic	Yes+ topical analgesic	No	Decrease in corticosterone, ~120 ng/ml after fMRI (D5)	Resting state fMRI
Febo, 2011	Sprague-Dawley	Quadrature surface coil (transmit/receive)	Cylindrical head holder	Body tube, limbs free	20 min to a maximum of 50 min, 5 days	No, but topical analgesic	Yes+ topical analgesic	No	–	fMRI, stress-evoking stimulus
Tsurugizawa et al., 2012	Sprague-Dawley	Surface coil compatible to head fixation mount	Head fixation acrylic mount	Rubber bands	30 min on first day and 90 min from thereon, 5 days	Head fixation	No	Yes	–	fMRI, reward anticipation
Brydges et al., 2013	Lister hooded rats	Single loop, surface coil	Cylindrical head holder	Body tube	90 min/day, 3 days	No	Yes	No	–	fMRI, conditioned fear circuitry
Kenkel et al., 2016	Sprague-Dawley	Quadrature volume coil (transmit/receive)	Head holder	Body tube	30 min/day, 4–5 days	Yes	No	No	–	fMRI, reward processing
Chang et al., 2016	Sprague-Dawley	Surface coil compatible to head fixation mount	Head fixation mount	Snuggle sack	30 min, 8–10 days	Head fixation	No	No	Decrease in corticosterone ~1,200 ng/ml after fMRI	Resting state fMRI, stimulus fMRI (air puff)

The existing awake imaging methods, however, have several shortcomings with regard to both technical feasibility and animal welfare. Many of these approaches require dedicated beds and/or receiver coils, with poor filling factors, making the replication of protocols by others practically impossible. Additionally, the use of possibly painful ear bars, the lack of ear protection, the lack of soft padding materials, and the use of long non-progressive habituation times (Table 1) may induce major acute pain or long-term stress.

In the present work, we aimed to avoid several of these shortcomings by implementing novel ideas and adopting known best practices to create a significantly improved approach for awake fMRI. We designed a 3D printable restraint kit compatible with a common rat bed type and standard RF-coil with good filling factor. In the kit design, we aimed to take the comfort of animal into account, to reduce the amount stress experienced by the animal, and to minimize the required habituation period, while still maintaining robust fixation of the head. Indeed, the physiologic markers of stress indicate that fully awake rats were able to adapt to the measurement environment in 4 days, showing very low peak concentrations of plasma corticosterone throughout the habituation period. Additionally, we show that by using light sedation, the habituation protocol could be shortened to 2 days. After the 4-day habituation period, the RSFC pattern of lightly sedated rats better resembled the pattern obtained under the awake state than that under an anesthetized state. Thalamo-cortical connectivity, however, was significantly suppressed in the lightly sedated state compared with the awake state.

### 3D printed restraint kit

The 3D printed restraint kit and protocol described here was robust and easy to use during the *in vivo* experiments. The initial preparations and fixation of the rat to the bed typically took less than 10 min under isoflurane anesthesia. The restraint kit was confirmed to be suitable for a wide range of rat sizes, as rats weighting from 200 to 480 g were tested in the pilot experiments. Because no dedicated surface coil or animal bed is required, our protocol provides an affordable option for any awake rat fMRI study design. We provide the restraint kit template (STL-file, Supplementary Material) for public use and for further modifications.

### Movement during fMRI

In fMRI studies, it is crucial that patients or animals remain motionless during the imaging for acquisition of artifact-free data, as movement can compromise the fMRI analysis (Power et al., 2012, 2014). In our habituation protocol, rats adapted quickly to the restraint environment and movement decreased rapidly. Importantly, the occasional lower body movement did not typically affect the head position, indicating robust fixation achieved by our restraint kit.

Furthermore, we assume the 3D-printed kit can be helpful to reduce head motion also in lightly sedated animals, as our 3D-printed kit has more fixation points compared to a standard rat head fixation setting and we do not use ear bars causing possible pain in ear canals.

Our analyses indicated that the awake rats, as expected, tended to move more frequently than the lightly sedated and anesthetized rats. Typically, head movement of the awake rats occurred in vertical Y-direction, which seemed to be due to shifts in the teeth positioning. Nevertheless, the amount of movement of awake rats in the present study was relatively equal to that of human movement in a clinical fMRI (Power et al., 2012, 2014). Based on the results reported by Power et al. (2012, 2014) we estimated the mean position change relative to voxel size (4 × 4 × 4 mm) to be 4–10% in humans, while in our rat experiments position change was 2.8 ± 0.2% relative to voxel size (0.391 mm shortest dimension).

### Stress

Based on the observed decreases in the stress indicators (plasma corticosterone level, movement, and heart rate), the custom-made restraint kit with the applied habituation protocol was well-suited for the acclimatization of both awake and lightly sedated rats.

The plasma corticosterone concentration, perhaps the most reliable stress marker, returned to the baseline level by habituation day 4 in the awake group. Similar trend was observed already by habituation day 2 in the lightly sedated group. Generally, corticosterone concentrations remained surprisingly low even after habituation day 1, as several groups report concentrations in the range of 240–550 ng/ml on the first habituation day (King et al., 2005; Chang et al., 2016). In our study, the corresponding value was 162 ng/ml in the awake group. This observation strongly indicates that the animals were more gently introduced to the measurement environment compared with several previous reports.

Moreover, movement, as an indicator of adaptation, decreased rapidly during the habituation period to very low levels compared with previous reports (King et al., 2005; Chang et al., 2016). Movement of the brain in our study was in the range of 0–350 μm, while previous studies reported movement in the range of 0–550 μm or 0–1,300 μm (King et al., 2005; Chang et al., 2016).

Heart rate decreased to close to 400 beats per minute in awake animals after the 4-day habituation period. This is considered to be within the normal heart rate range in adult Wistar rats during the active day phase (Zhang and Sannajust, 2000).

Among the awake and lightly sedated groups, body weight did not decrease during the habituation weeks, which is in line with other observations, suggesting only minor stress induced by the habituation protocol. In the awake group, weight increased in a normal manner already over the weekend, further supporting the normal behavior.

Breathing rate and movement pointed to clear differences in the brain state between rats in the awake and lightly sedated groups. Breathing rates of lightly sedated rats were notably lower than those of awake rats on the MRI day 1. Movement was already minimal in lightly sedated rats from the starting day and also differed from that of awake rats on the MRI day 1.

In both animal groups, plasma corticosterone concentrations and movement levels tended to increase slightly on the imaging day compared with the 4th habituation session, indicating a small difference between the habituation and scanning conditions. Certain properties of the MRI device, such as possible sounds that may mimic ultrasonic communication between rats (>20 kHz), could not be reproduced in our mock scanner. Furthermore, small mechanical vibrations coming from the gradient set could not be mimicked in the habituation environment. Nevertheless, the slight increases in the stress indicators did not reach statistical significance.

In addition to acute stress, rats can develop chronic stress after the habituation protocol. Low et al. (2016) demonstrated that long-lasting elevated plasma corticosterone concentrations, reduced nociceptive behavior, and increased activation of the central amygdala are observed following commonly applied habituation protocols (30 min, 3 days). Therefore, chronic stress can mask or compromise fMRI results, especially those obtained at later time-points. To address this issue, we evaluated depression- and anxiety-like behavior in habituated rats with well-known open-field and sucrose preference behavioral tests (Brenes Sáenz et al., 2006). Our results suggested no differences between habituated and control rats in locomotor activity, rearing, or center time in the open-field, or in the sucrose preference test, which speaks against major anxiety or depression-like behavior due to the habituation. In contrast, the habituated rats groomed more often than the control rats. This could indicate that the habituated rats felt more comfortable, as they were used to handling and experiencing new environments, thus making their adaptation faster. However, we recognize that the relatively small group sizes in behavioral tests may have hindered the detection of potential differences.

### Habituation time

The shortest required training period for awake and lightly sedated rats based on the measured indicators for stress was estimated to be 4 and 2 days for the awake and lightly sedated rats, respectively, to achieve a low amount of movement and low corticosterone concentrations. Additionally, heart rate decreased significantly in the awake group during the 4-day period. Importantly, our RSFC data show similar connectivity at the end of the first and second week of habituation. Thus, the additional week of habituation yielded no additional benefit for connectivity analysis.

A protocol in which rats are completely restrained for a prolonged time, i.e., several hours, is widely used to study chronic stress (Chiba et al., 2012; Stepanichev et al., 2014). To avoid chronic stress, habituation sessions in different awake MRI studies typically last only in the range of 30–90 min. To elaborate this protocol further, in our study, the restraint session times started as a very short period of 15 min and progressively increased up to a maximum of 45 min on the day 4.

### Resting state connectivity

Overall, the measured functional connectivity differed significantly between brain states. Even with a sub-anesthetic dose of isoflurane, the connectivity was significantly modulated compared with awake rats.

Strong connectivity across the cortical regions in the 1.3% isoflurane group is likely due to the anesthesia-induced burst suppression activity, which is characterized by alternation of silent brain states with almost no electrical activity (suppression) and active states with high-amplitude activity (bursts) (Derbyshire et al., 1936). Isoflurane induces burst suppression activity at a wide range of doses (e.g., 1.25–2.0%) (Hudetz and Imas, 2007; Vincent et al., 2007; Liu et al., 2013). The cycling between silent and active states is directly reflected by the hemodynamics and therefore is also visible in the fMRI BOLD signal (Liu et al., 2011, 2013). This cycling is likely to induce strong cortico-cortical and cortico-striatal correlations. Similar strong cortico-cortical or cortico-striatal connectivity in rats under high isoflurane anesthesia has been also observed previously (Williams et al., 2010; Liu et al., 2011, 2013; Kalthoff et al., 2013). When the cortical and cortico-striatal burst suppression synchronization is the most dominant feature in the brain, it is evident that the intrinsic connectivity is heavily masked.

The effect of burst suppression was also evident in the DMN in the 1.3% isoflurane group. While DMN is generally suppressed under anesthesia (Deshpande et al., 2010; Liu et al., 2015), brain regions associated with the DMN exhibited increased connectivity under 1.3% isoflurane anesthesia compared with awake rats. By contrast, isoflurane anesthesia induced heavy disruption of cortico-subcortical connectivity, mainly affecting connections between the cortex and medial thalamus. As the thalamus works as a key hub for controlling sensory information flow to the cortex, disrupted thalamo-cortical connectivity is considered to be the central mechanism regulating consciousness (Nallasamy and Tsao, 2011).

In the lightly sedated group, there were no abnormally high cortico-striatal correlations indicating burst suppression activity, which is consistent with previous reports (Liu et al., 2013), enabling more detailed analysis of intrinsic connectivity. Indeed, the FC matrices and maps in the cortex and striatum were fairly similar between the lightly sedated and awake rats, although global connectivity tended to be slightly suppressed. Suppression of connectivity most likely originates from reduced excitatory activities or increased inhibitory activities (Franks, 2008) induced by the low isoflurane dose. Additionally, we observed significantly suppressed connectivity in three medial thalamo-cortical connections in the seed-based connectivity analysis. In the voxel-wise analysis, however, this phenomenon was not detected after correcting for multiple comparisons. In summary, light sedation protocol allows two-times faster habituation protocol, but its drawbacks are the anesthesia-induced effects on RSFC that appear to be emphasized in the thalamo-cortical connectivity.

### Effect of physiology and methodologic considerations

In this study, we used a spin echo EPI sequence to study resting state networks. We have used spin echo EPI (SE-EPI) to have comparable results obtained from our previous EEG/fMRI studies which are conducted using SE-EPI to reduce susceptibility artifacts caused by electrodes. However, most of the fMRI studies today are conducted with a gradient echo EPI. While fMRI BOLD contrast in spin echo EPI has more specificity to small capillaries, gradient echo EPI is more sensitive to larger venules giving more detectable fMRI contrast (Lee et al., 1999). This can potentially influence comparison of rsfMRI maps obtained from studies implementing gradient echo EPI sequences. It is also worth noting that the current study was conducted with a 7T magnet, and with the higher magnetic fields, motion related artifacts can increase.

Because of local availability, Wistar rats were used in this study instead of Sprague-Dawley rats, despite SD rats are known to be less anxious and easier to habituate (Rex et al., 2004). Moreover, despite from results indicating female rats being more adaptable to restraint (Albonetti and Farabollini, 1992), we used male Wistar rats instead of female rats as the majority of biomedical research is conducted using male rats to avoid possible bias caused by hormonal rhythm variations.

As physiologic parameters such as breathing and heart rates differ between awake, lightly sedated, and anesthetized animals, they must be taken into account in the connectivity analysis. For this purpose, both high-pass and low-pass filters were used for the BOLD signals. A low-pass filter at 0.15 Hz was used to minimize the effect of physiologic noise, like respiratory and heart rate artifacts, on the BOLD signal. The high-pass filter at 0.01 Hz reduces the possible false-positive correlation of extremely low fluctuations of the BOLD signal originating from, e.g., hardware-related drifts. Nevertheless, the low sampling rate (0.5 Hz) does not allow for the complete removal of physiologic noise, and the effect on the connectivity analysis remains unclear.

Spontaneous behavioral changes, such as changes in eye state (open vs. closed), variations in respiratory and olfactory activity of awake animals can influence dynamic connectivity (Liu and Duyn, 2013; Di and Biswal, 2015). During the habituation sessions, rats kept their eyes open and blinked normally. Once rats woke up from the initial anesthesia, breathing rate remained mostly stable during the habituation and fMRI sessions. In addition, based on respiratory rates, rats did not spontaneously fall asleep (possible due to slight stress and noise) during the scanning. Spontaneous olfactory stimulation was controlled by cleaning the animal beds after imaging each rat, and the scanner room was kept clean from other animal odors. In this study, we used standard EPI sequence causing significant sound pressure. The current experimental setup can be clearly improved by using pulse sequences with sound optimized gradient wave form design (Hutter et al., 2018) or radial acquisition (Lehto et al., 2017).

As rats were initially anesthetized and prepared under isoflurane, we cannot completely ignore the possible residual effects of isoflurane on functional connectivity in awake animals. The effect of isoflurane is short, however, and it is eliminated rather quickly from the body as rats usually wake within 2–4 min after the cessation of isoflurane, depending on the dose and duration. Also, isoflurane was turned off already during the shimming stage. Therefore, animals had several minutes to wake up from the anesthesia before the functional scanning started. Furthermore, the first minute from each functional dataset was excluded from analysis.

Finally, in the sub-cohort open field and sucrose preference studies, the relatively small animal numbers could possibly prevent the detection of small differences between groups, however, it rules out possible robust long-term anxiety or depression caused by stress.

## Summary and conclusion

We present a novel and easily implemented approach for awake rat fMRI. Our method introduces a 3D printed rat restraint kit compatible with a standard Bruker rat MRI bed, and quadrature surface receiver and transmitter coils. By using this kit and a modified habituation protocol, we were able to perform low-stress RSFC studies in fully awake rats. Based on measured physiologic markers for stress and the amount of movement, a 4-day acclimatization period was sufficient for awake rats, and the habituation protocol can be shortened to 2 days when 0.5% isoflurane is used. Additionally, we demonstrated that 1.3% isoflurane anesthesia in rats markedly affected brain connectivity compared with that in the awake state, and that the effect was significant, albeit less marked, for cortico-thalamic connections when 0.5% isoflurane was used. Overall the proposed approach is likely to make awake fMRI studies in rat more common in future, and thus increase the reliability and translatability of the results from rat fMRI studies.

## Author contributions

PS and JP contributed in data collection and analysis. RS contributed in data analysis. KJ contributed in the 3D printing process. HT was consulted in the behavioral tests. OG contributed in the study design. PS contributed in writing of the manuscript and all authors took part in reading and revision of the manuscript.

### Conflict of interest statement

The authors declare that the research was conducted in the absence of any commercial or financial relationships that could be construed as a potential conflict of interest.
