# Supplementary material for: Awake Rat Brain Functional Magnetic Resonance Imaging Using Standard Radio Frequency Coils and a 3D Printed Restraint Kit
Source: Front Neurosci. 2018 Aug 20;12:548. doi: 10.3389/fnins.2018.00548 (PMC6109636; doi:10.3389/fnins.2018.00548)

**Supplementary Material**

**Awake rat brain functional magnetic resonance imaging using standard radio frequency coils and a 3D printed restraint kit**

Petteri Stenroos^1^, Jaakko Paasonen^1^, Raimo A. Salo^1^, Kimmo Jokivarsi^1^, Artem Shatillo^2^, Heikki Tanila^1^, Olli Gröhn^1*^

^1^Kuopio Biomedical Imaging Unit, A.I.V. Institute for Molecular Sciences, University of Eastern Finland, Kuopio, Finland

^2^Charles River Discovery Research Services Finland Oy, P.O. Box 1188YTJ, FI-70211 Kuopio, Finland

*Correspondence:

Prof. Olli Gröhn

olli.grohn@uef.fi

1. **Supplementary video**

<https://studentuef.sharepoint.com/sites/AwakefMRI/Shared%20Documents/Forms/AllItems.aspx>

For further request, please contact either the corresponding author (olli.grohn@uef.fi) or the first author (petteri.stenroos@uef.fi).

1. **Rat restraint kit**

<https://studentuef.sharepoint.com/sites/AwakefMRI/Shared%20Documents/Forms/AllItems.aspx>

For further request, please contact either the corresponding author (olli.grohn@uef.fi) or the first author (petteri.stenroos@uef.fi).

1. **Supplementary figures**

**Suppl. Figure 1. Regions of interests (ROIs) used in the connectivity analysis.** 12 ROIs (A) were used as seed regions in the whole-brain analysis and 7 ROIs (B) were used in the default mode network analysis. Distance from bregma is marked in each brain slices.


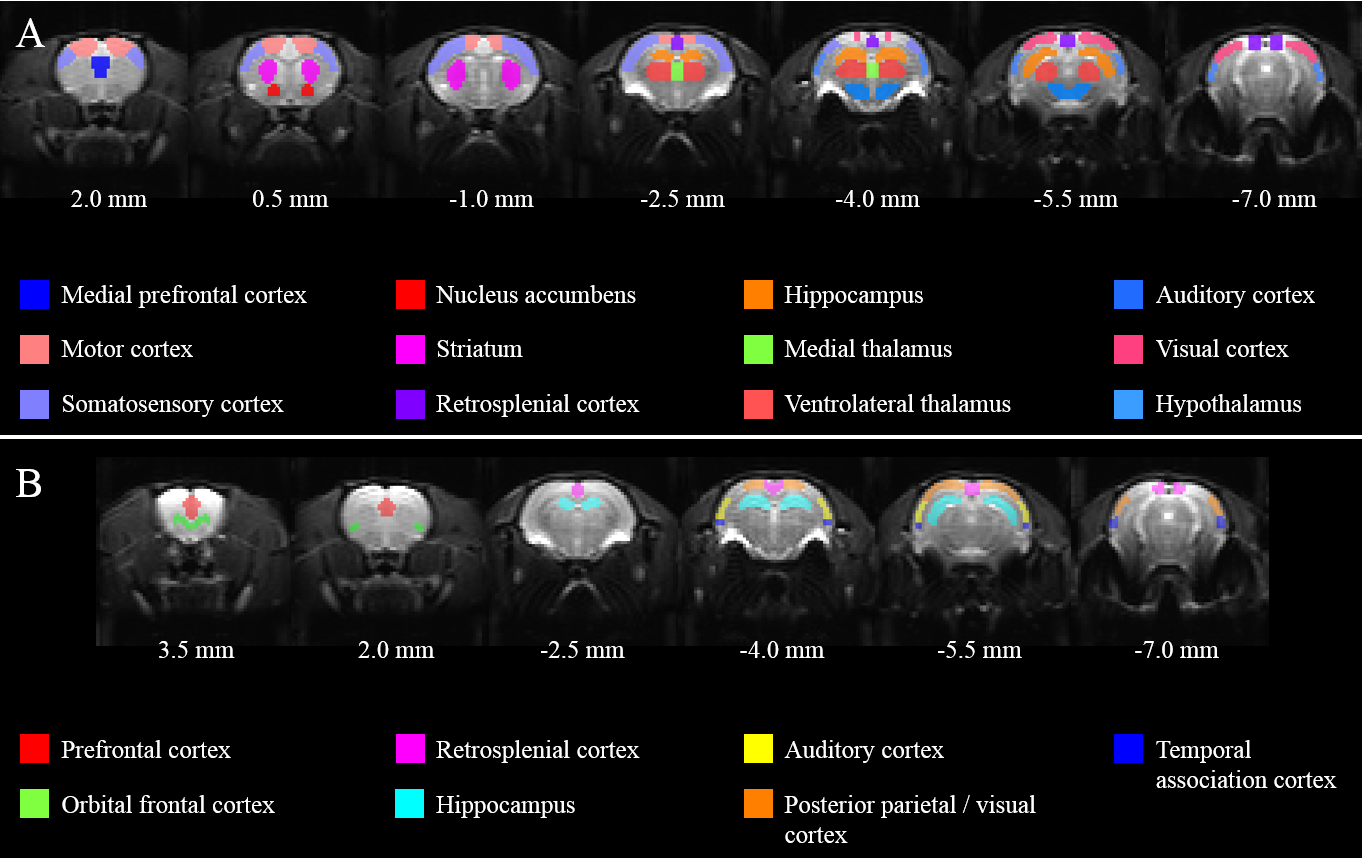

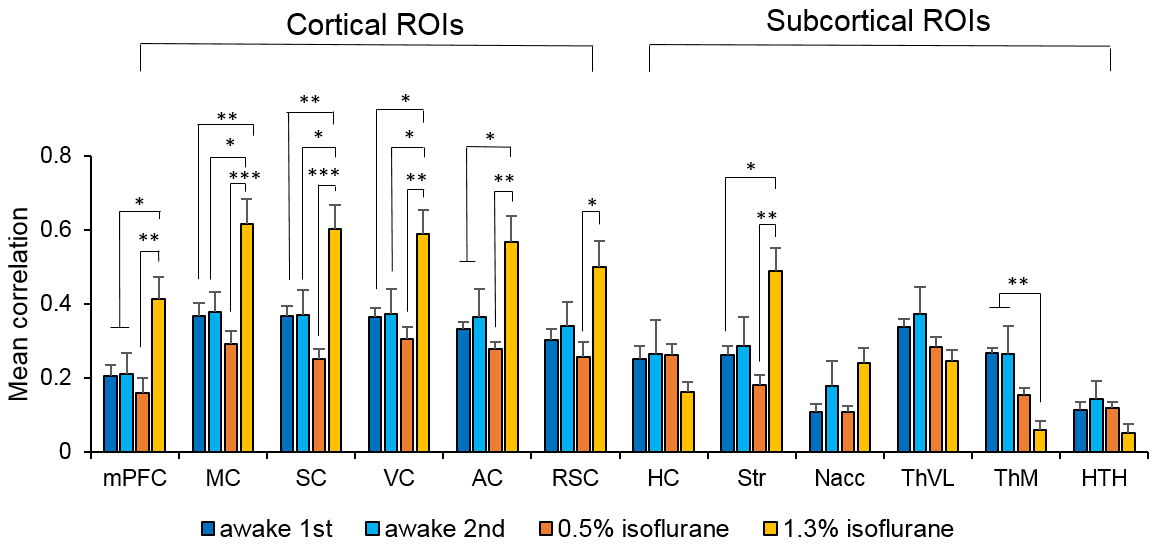


**Suppl. Figure 2. Region specific mean (+ SEM) functional connectivity between 12 regions of interest in 1^st^ and 2^nd^ awake time points, 0.5% isoflurane and 1.3% isoflurane groups.** Mean correlation values of each ROI to all other ROIs is presented. Statistical testing was done by one-way ANOVA and Tukey´s multiple comparison post-hoc test, *p<0.05, **p<0.01, ***p<0.001. AC, auditory cortex; HC, hippocampus; HTH, hypothalamus; mPFC , medial prefrontal cortex; ThM, medial thalamus; MC, motor cortex; Nacc, nucleus accumbens; RSC, retrosplenial cortex; SC, somatosensory cortex; Str, striatum; ThVL, ventrolateral thalamus; VC, visual cortex.


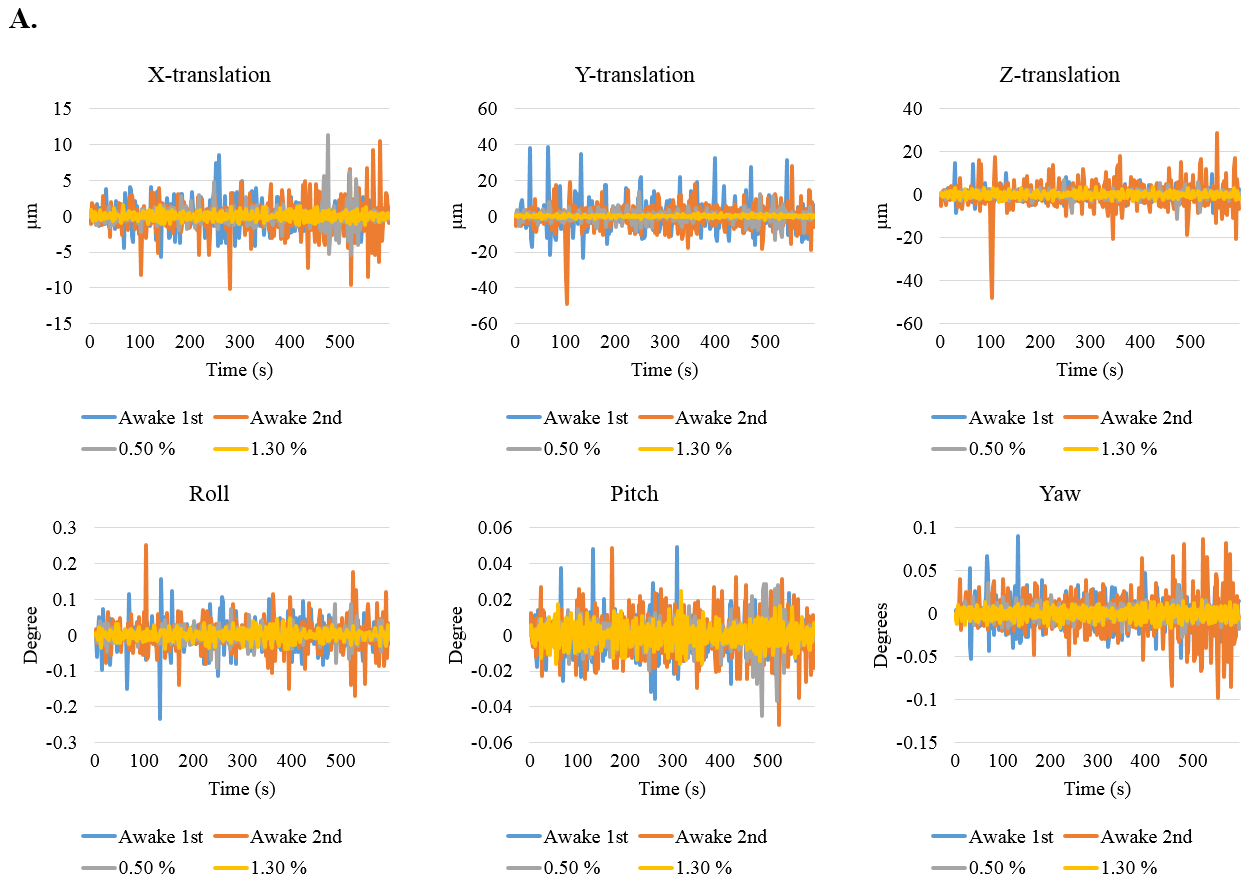

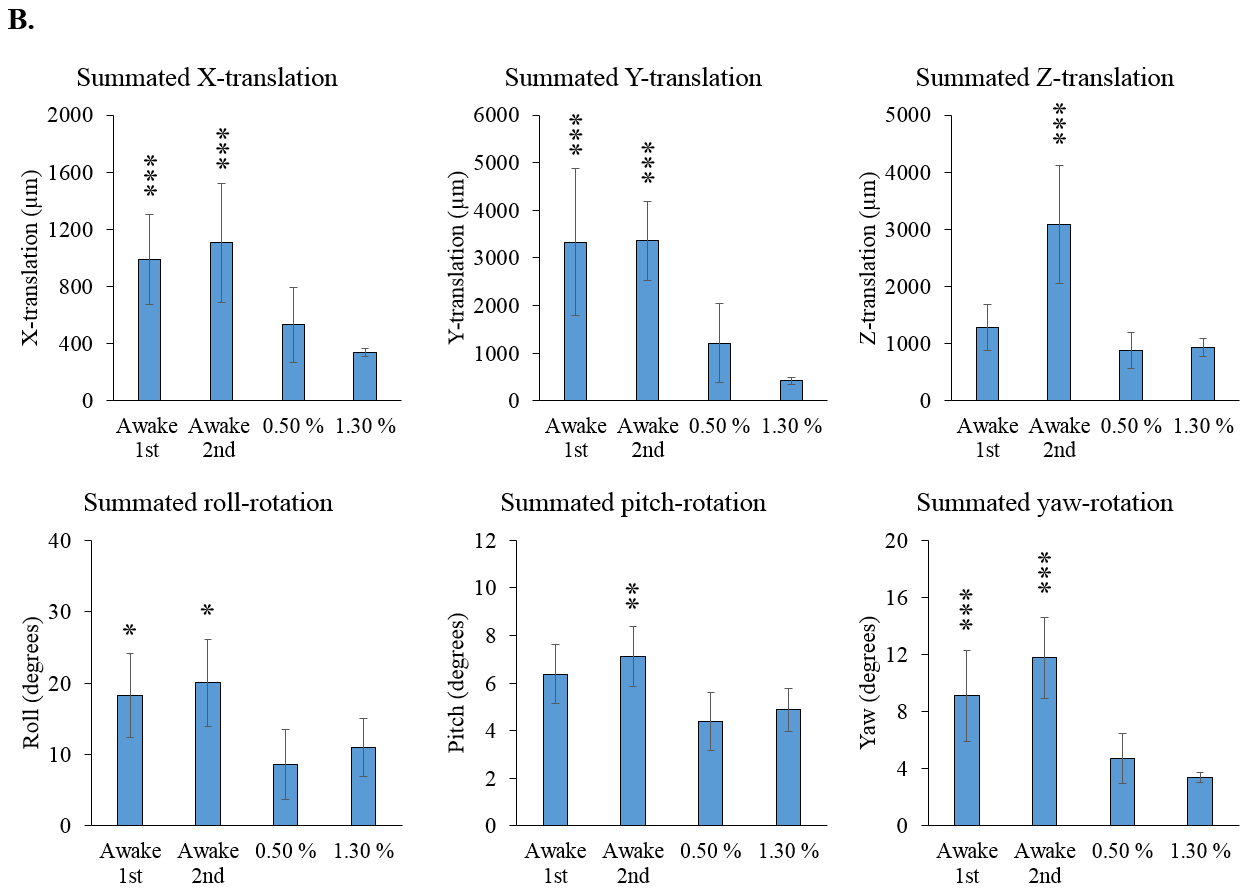


**Suppl. Figure 3. Average (A) and summated (B) translational and rotational movement in 1^st^ and 2^nd^ awake time points, 0.5% isoflurane and 1.3% isoflurane groups.** Average and summated x, y, z –translational (µm) and rotational (degrees) movement were calculated during 10 min fMRI sessions. Statistical testing was done by one-way ANOVA and Tuckey´s multiple comparison post-hoc test, *p<0.05, **p<0.01, ***p<0.001. Significances to 1.3% isoflurane group is reported.

**Suppl. Figure 4. Maximum displacement of the head from the initial position (mean + SEM) of rats during fMRI in 1^st^ and 2^nd^ awake time points, 0.5% isoflurane and 1.3% isoflurane groups.** Statistical testing was done by one-way ANOVA and Tukey´s multiple comparison post-hoc test, **p<0.01. Significances to 1.3% isoflurane group is reported.


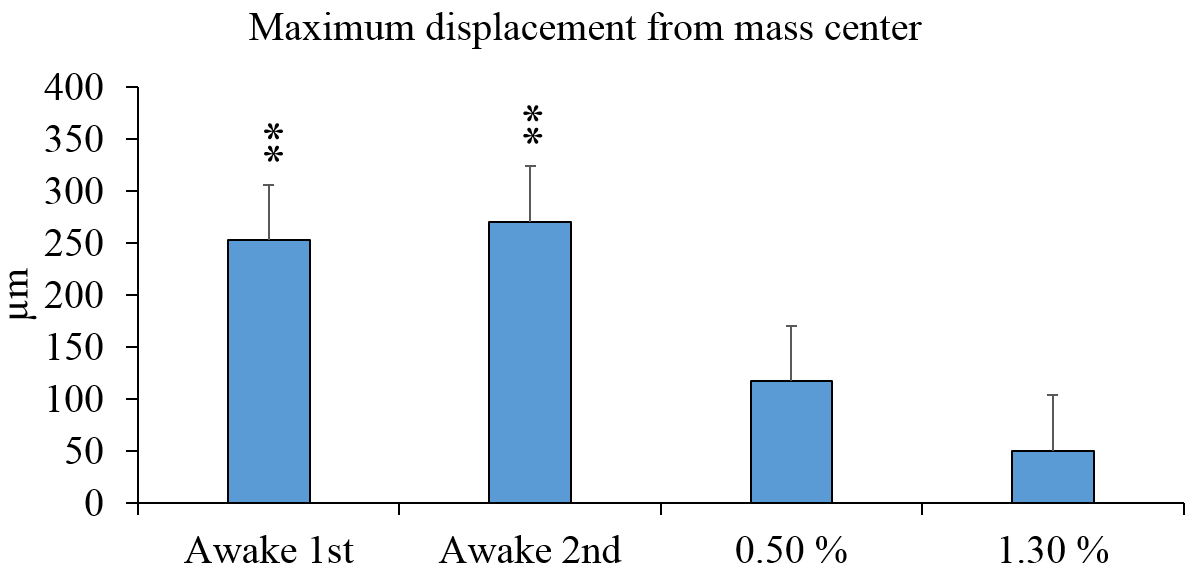


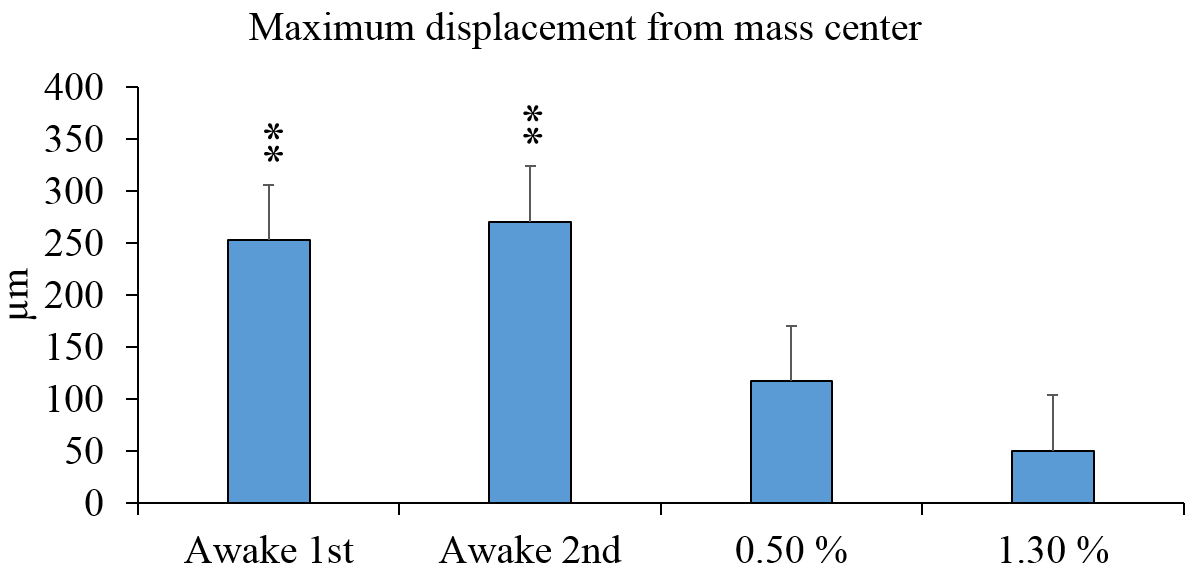


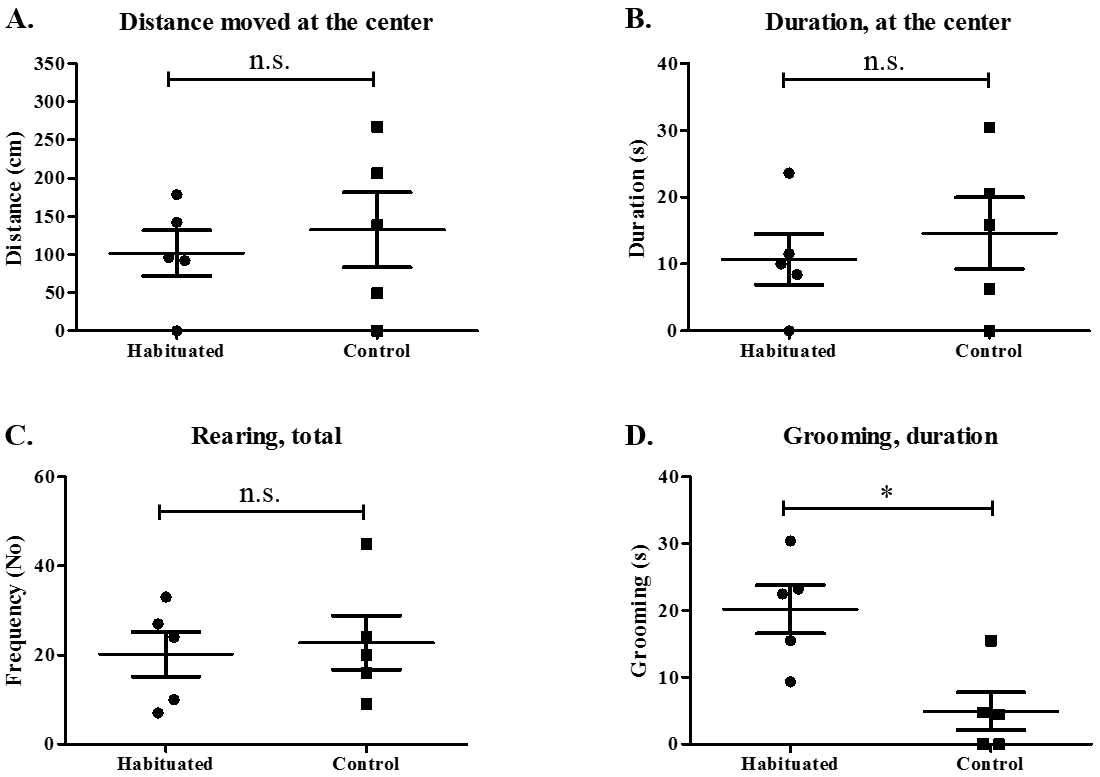


**Suppl. Figure 5.** **Open field result from the subgroup of habituated and control rats.** Results of movement distance (cm) (A), duration (s) (B), rearing (C) and grooming behavior (D) during the 10min acquisition. Statistical testing was performed with a two-sample, two-tailed t-test. n.s., no statistical significance, *p<0.05.

**Suppl. Figure 6. A sucrose preference of the subgroup of habituated and control rats.** Preference demonstrates percentage of consumed sucrose water compared to total consumed water during the 2-day period. Statistical testing was performed with a two-sample, two-tailed t-test. n.s., no statistical significance.


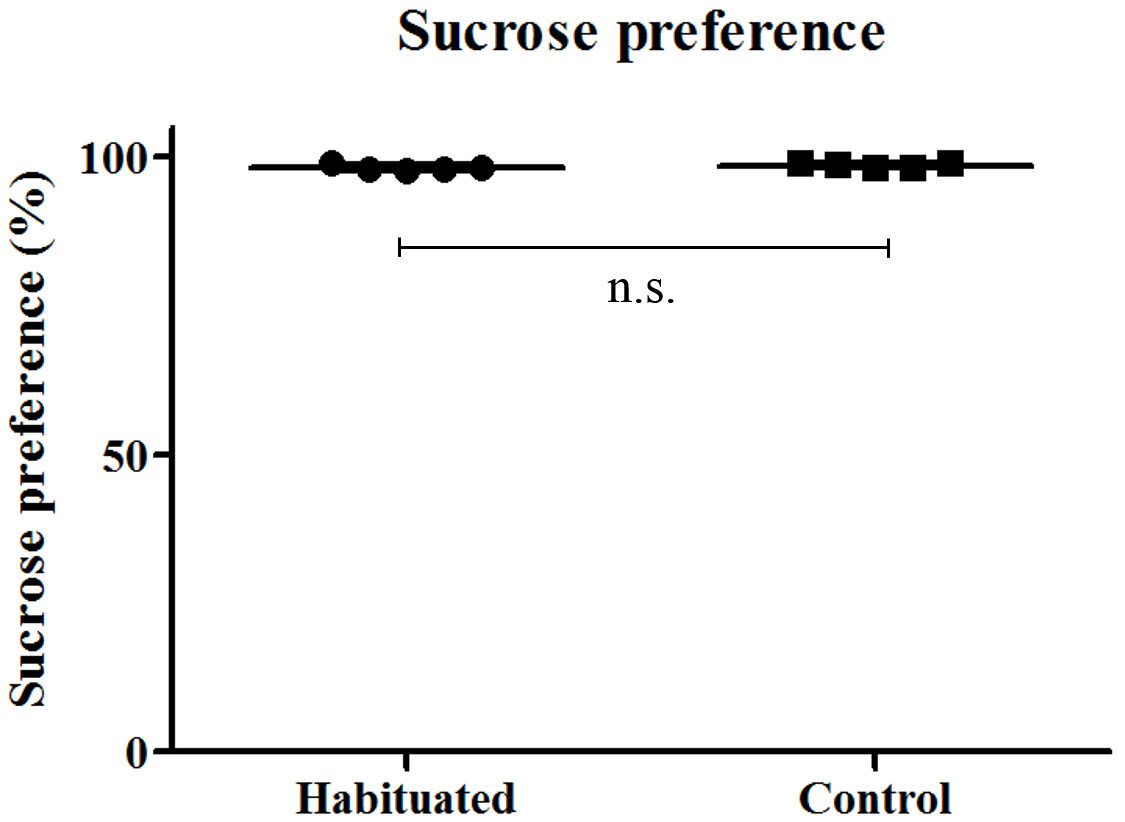


**Suppl. Figure 7.** **Raw echo planar imaging time series and mass center translation values obtained from primary somatosensory cortex from two representative awake rats.** 25 minutes of fMRI data were collected from each rat (A, D). 10-minute periods (red boxes), where rats moved the least, were selected (B, E) for data preprocessing and analysis. Corresponding mass center displacement values, given by SPM8, are shown in C and F.


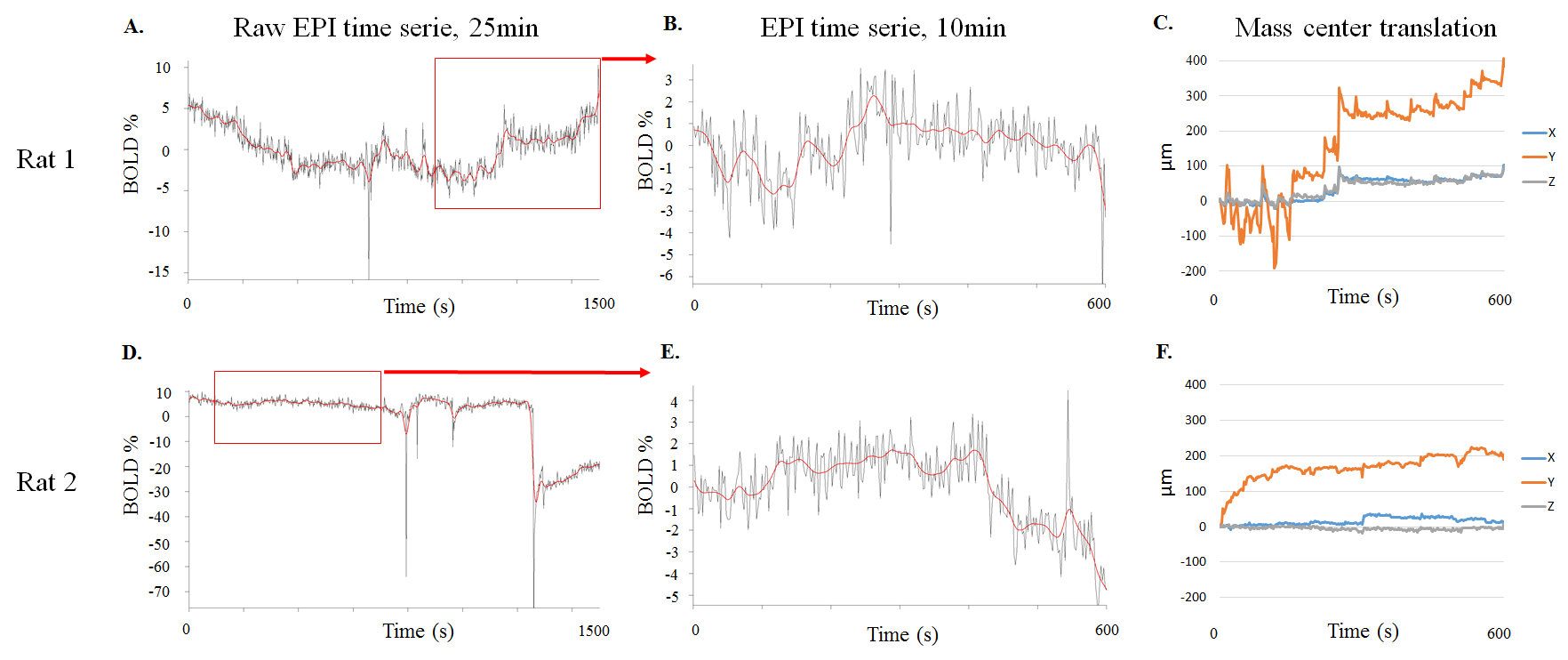

Supplement: Supplementary file 1 [file Data_Sheet_1.DOCX]
